# Relationships between psychosocial outcomes in adolescents who are obese and their parents during a multi-disciplinary family-based healthy lifestyle intervention: One-year follow-up of a waitlist controlled trial (Curtin University’s Activity, Food and Attitudes Program)

**DOI:** 10.1186/s12955-016-0501-z

**Published:** 2016-07-07

**Authors:** Ashley A. Fenner, Erin K. Howie, Melissa C. Davis, Leon M. Straker

**Affiliations:** School of Psychology and Speech Pathology, Curtin University, GPO Box U1987, Perth, WA 6845 Australia; School of Physiotherapy and Exercise Science, Curtin University, GPO Box U1987, Perth, WA 6845 Australia

**Keywords:** Obese, Intervention, Adolescent psychosocial, Parent psychosocial, Multi-disciplinary

## Abstract

**Background:**

Limited studies have investigated relationships in psychosocial outcomes between adolescents who are obese and their parents and how psychosocial outcomes change during participation in a physical activity and healthy eating intervention. This study examined both adolescent and parent psychosocial outcomes while participating in a one - year multi-disciplinary family-based intervention: Curtin University’s Activity, Food, and Attitudes Program (CAFAP).

**Methods:**

Following a waitlist control period, the intervention was delivered to adolescent (*n* = 56, ages 11–16) and parent participants over 8 weeks, with one-year maintenance follow-up. Adolescent depression and quality of life, family functioning, and parent depression, anxiety, and stress were assessed at six time points: baseline and prior to intervention (e.g., waitlist control period), immediately following intervention, and at 3, 6, and 12 months post-intervention. Relationships between adolescent and parent psychosocial outcomes were assessed using Spearman correlations and changes in both adolescent and parent outcomes were assessed using linear mixed models. Changes in adolescent psychosocial outcomes were compared to changes in behavioural (physical activity and healthy eating) and physical (weight) outcomes using independent samples t-tests.

**Results:**

The majority of psychosocial outcomes were significantly correlated between adolescents and parents across the one-year follow-up. Adolescent depression, psychosocial and physical quality of life outcomes significantly improved before or following intervention and were maintained at 6-months or one-year follow-up. Parent symptoms of depression, anxiety, and stress were reduced during waitlist and primarily remained improved. Changes in adolescent psychosocial outcomes were shown to be partially associated with behavioural changes and independent of physical changes.

**Conclusions:**

Adolescents in CAFAP improved psychosocial and physical quality of life and reversed the typical trajectory of depressive symptoms in adolescents who are obese during a one-year maintenance period. CAFAP was also effective at maintaining reductions in parent symptoms of depression, anxiety, and stress demonstrated during the waitlist period.

**Trial Registration:**

The trial was registered with the Australian and New Zealand Clinical Trials Registry (No. 12611001187932).

## Background

The prevalence of adolescent overweight and obesity has increased dramatically over the past three decades [1]. Higher rates of anxiety and depressive symptoms, as well as poorer quality of life, have been identified as consequences of adolescent obesity [2, 3]. Whilst physical consequences of adolescent obesity tend to be observed in the long-term [4], psychosocial consequences of obesity such as depressive symptoms and quality of life, are believed to be more immediate [5].

In addition to compromised psychosocial states in adolescents who are obese, evidence suggests that parents of these adolescents also experience elevated symptoms of depression, anxiety, and stress [6, 7]. These poorer psychosocial states are likely to influence parents’ ability to provide optimal environments for promoting positive psychosocial outcomes and engagement in healthy lifestyle behaviours for adolescents [8]. Further, poorer family functioning is positively associated with adolescents’ weight status [9], and more impaired psychosocial functioning in adolescents [10] and parents [11]. Therefore the psychosocial status of parents of adolescents who are obese may be an important factor when designing adolescent obesity interventions.

Best-practice recommendations suggest that treatment for adolescent obesity should be family-based and target behaviour change alongside psychosocial determinants in both adolescents and parents. Despite the evidence of psychosocial issues for parents of adolescents who are obese, intervention studies have primarily focused on adolescent behavioural and physical outcomes, with minimal attention to psychosocial outcomes in adolescents and their parents [12], and no investigations of the associations between adolescent and parent psychosocial outcomes across pre- and post- intervention periods. In addition, despite the well-established need for a minimum of one-year follow-up to determine meaningful improvements following intervention [13], few studies have explored the maintenance of changes following multi-disciplinary family-based interventions targeting adolescents [14–17]. Among these limited studies, promise has been shown in relation to improvements in global self-worth, well-being, self-concept, and body satisfaction [15–18]. Depressive symptoms have however not been assessed and quality of life findings have been confounded by the inclusion of children (e.g. 8–17 years of age) [19], limited parental involvement (e.g., 2 sessions) [14, 20], and parent content delivered by a single health practitioner (e.g., dietician) [21].

Curtin University’s Activity, Food and Attitudes Program (CAFAP) was a community-centred family-based intervention delivered by teams of multi-disciplinary health professionals that targeted the promotion of healthy lifestyle behaviours within the context of enhancing adolescent and parent well-being. Evaluation of behavioural changes (physical activity, sedentary time, and healthy eating) showed small significant improvements immediately following the 8-week intervention and some of the changes were maintained up to one year, with non-significant reductions in physical outcomes (BMI) [22]. The intervention also resulted in changes in perceived parental need-supportive behaviours and adolescent autonomous motivation to change behaviour (physical activity and healthy eating), with improvements in adolescent autonomous motivation partly mediated by improvements in adolescent health-related quality of life [23].

The current analysis aimed to: 1) evaluate the relationship between adolescent and parent psychosocial outcomes, 2) examine psychosocial changes over the course of the intervention and 12-months follow-up, and 3) examine how adolescent psychosocial changes related to behavioural and physical changes. It was hypothesised that: 1) adolescent quality of life outcomes (psychosocial, physical, and health-related) would be negatively related to parent psychosocial outcomes (depression, anxiety, stress) and positively related to family functioning scores, with adolescent depressive symptoms showing an inverse relationship, 2) adolescent and parent psychosocial outcomes would improve with the intervention and improvements would remain across the one-year maintenance period, and 3) improvements in adolescent psychosocial outcomes (depressive symptoms and psychosocial and physical quality of life) would be related to improvements in adolescent behaviours (physical activity and healthy eating) and physical status (BMI).

## Methods

### Study design

This study was part of a larger waitlist controlled trial [22, 24] using a staggered cohort design to balance seasonal effects. This design controls for threats to internal and external validity while not unethically withholding a valid treatment from adolescents. Participants completed baseline assessments, were waitlisted for one school term (three months), and completed pre-intervention assessments in order to provide a within-participant control period. Post-intervention assessments were completed immediately after the intensive 8-week intervention and at 3, 6, and 12 months after intervention conclusion, totalling six assessment points. During the one-year maintenance period adolescents received tapered phone call and text message support [25]. The trial was registered with the Australian and New Zealand Clinical Trials Registry (No. 12611001187932), and approved by Curtin University Human Research Ethics Committee (HR105/2011). Informed consent was obtained from all adolescents and parents prior to commencing participation.

### Participants

Adolescents and their parents were recruited through the health system, local schools, and the general community. Adolescents were eligible to participate if they were between 11 and 16 years of age, had greater than the 85th percentile on the Centers for Disease Control BMI-for-age growth charts [26], passed screening conducted by a medical practitioner, were willing to attend the intervention and all assessments, and were not obese due to a medical condition or undergoing treatment for a psychological disorder.

### Measures

#### Adolescent psychosocial outcomes

Depressive symptoms were measured using the Short Moods and Feelings Questionnaire (SMFQ) [27]. The 13-item scale is derived from the 34-item depression questionnaire (Moods and Feelings Questionnaire) [28] and assesses self-reported symptoms experienced in the preceding two weeks using a 3-point scale ranging from 0 (*not true*) to 2 (*true*), with higher scores reflecting more depressive symptoms. The SMFQ has high internal consistency [29] and has been validated in clinical and non-clinical samples [30].

Quality of life was measured using the Paediatric Quality of Life – Teen Report (PedsQL) [31]. The 23-item measure consists of generic core scales that encompass physical functioning, emotional functioning, social functioning, and school functioning. Adolescents indicated how much of a problem each item had been in the past month using a 5-point scale ranging from 0 (*never a problem*) to 4 (*almost always a problem*). Items are reverse scored and linearly transformed to a zero to 100 scale (0 = 100, 1 = 75, 2 = 50, 3 = 25, 4 = 0), so that higher scores indicate better quality of life. Scale scores are used to derive measures of psychosocial and physical quality of life, and are summed to provide an overall assessment of health-related quality of life. Changes in subscale scores are reported in the current study to align with assessment of the relationship to behavioural and physical changes. Changes in overall scores have been previously reported [32], and are reported here in relation to parent psychosocial outcomes. The measure has been demonstrated to have good reliability and validity [33].

#### Parent psychosocial outcomes

The 21-item version of the Depression Anxiety and Stress Scale (DASS-21) [34] was used to measure parent symptoms of depression, anxiety, and stress. Parents indicated the extent to which they had experienced each symptom in the past week using a 4-point scale ranging from 0 (*does not apply to me*) to 3 (*applies to me most of the time*), with higher scores indicating greater symptomatology. The DASS-21 has been shown to be a reliable and valid measure in non-clinical samples [35].

Family functioning was assessed using the 12-item General Functioning scale of the McMaster Family Assessment Device (FAD-GF) [36]. Parents were asked to indicate their family’s ability to respond to stressful events using a 4-point scale ranging from 1 (*strongly disagree*) to 4 (*strongly agree*), with higher scores indicating more family dysfunction. The FAD-GF has been shown to have good reliability and validity [37].

#### Intervention

The intensive 8-week (two hour bi-weekly sessions) intervention was implemented in community settings (e.g., neighbourhood community centres) by locally recruited multi-disciplinary teams (physiotherapists/exercise physiologists, dieticians, and psychologists) across three waves in one rural and two metropolitan locations in Western Australia. CAFAP was based on self-determination theory [38] and goal setting theory [39], with a focus on enhancing adolescents’ autonomous motivation for healthy lifestyle behaviours and parents’ autonomous motivation to support adolescents’ behaviour changes. Instructors and parents were trained in using need-supportive behaviours, and key messages focused on enjoyment of healthy lifestyle behaviours rather than weight loss. During the first hour of each session, adolescents participated in physical activity circuits led by physiotherapists/exercise physiologists instructors, while parents received education information (i.e., training in need-supportive behaviours and goal setting to support adolescents, understanding adolescence, parenting styles, and food budgeting) as well as opportunities for sharing experiences. The second hour was jointly attended by adolescents and parents, and included topics such as setting goals, nutrition principles, practical strategies for behaviour change, overcoming barriers and problem solving, and family cohesion. Additional details of the study’s theoretical underpinnings, program rationale, and protocol have been reported elsewhere [24, 40].

### Statistical analyses

Histograms and q-q plots of dependent variables were examined for normality. Independent samples t-tests or ANOVAS were used to compare differences in baseline variables between waves, sites, and completers and those who dropped out either during the waitlist or maintenance period.

The relation of adolescent and parent psychosocial outcomes was assessed using Spearman correlation coefficients at baseline and across all time points. All statistical analyses were performed using Stata 13 (StataCorp LP, College Station TX, USA).

To assess changes in psychosocial outcomes during the intervention, linear mixed models were used with an exchangeable correlation structure to compare outcomes immediately following the intensive 8-week intervention and at 3, 6, and 12 month follow-up. Models included a random intercept for each participant to account for within-participant repeated measures. As the dependent variables deviated from normal distributions, sensitivity analyses using squared transformations of the health-related quality of life subscales and negative binomial regression for the SMFQ and DASS-21 were conducted. Residual plots were examined for appropriateness of the models and did not largely differ between alternate models. The magnitudes of trends were similar between alternative models, thus the linear regressions are presented for ease of interpretation. Standard errors were bootstrapped with 1,000 replications to adjust for slight deviations from normality.

Only participants who began the intervention (e.g., completed a minimum of two assessments) were included in the statistical models (*n* = 56). Within the linear mixed model, non-biased estimates were calculated for the missing values using likelihood-based estimation which uses information from surrounding responses and the covariance structure which is an appropriate method in studies with high levels of drop-out and drop-out bias [41]. For all participants across all time-points, the rate of missingness was 22% (see participant flow [22]). Additionally, sensitivity analyses with only those who completed the entire intervention and maintenance period were conducted to examine the robustness of the results.

A priori linear contrasts were used to compare mean point estimates at each assessment to point estimates at pre-intervention. Additionally, monthly rates of change were estimated for each assessment period to allow for adjustment in variations of time elapsed between assessment points. The rate of change during intervention (e.g., between pre-intervention and post-intervention) and maintenance periods (e.g., between post-intervention and 12-month follow-up) were compared to the rate of change during the waitlist control period (e.g., baseline to pre-intervention). No adjustments were made for multiple comparisons, rather 95 % confidence intervals are presented for all parameter estimates and *p*-values included where appropriate.

To explore the extent to which changes in psychosocial outcomes were associated with changes in behavioural (moderate and vigorous physical activity and junk food intake) and physical (BMI) outcomes, participants were divided into tertiles based on their improvements in scores on the SMFQ and psychosocial and physical quality of life subscale scores, from pre- to post- intervention and from pre- to 12-months post-intervention. Independent samples t-tests were then used to compare differences in behavioural and physical changes between the top and bottom tertiles in psychosocial outcomes for both periods of change.

## Results

### Participants

A total of 68 adolescent/parent pairs participated in entry assessments, and 56 completed both entry and pre-intervention assessments and began the intervention (see for participant flow [22]). There were no differences in adolescent and parent variables at baseline among participants who dropped out during the waitlist period, maintenance period (i.e., grouped across 3, 6, and 12 months), and those involved for the duration of the study (all p values > .05). Further, there were no significant differences between waves or sites in adolescent and parent-reported outcomes at baseline (see for participant characteristics [22]).

Adolescent participants included in analyses had a mean age of 13.9 ± 1.5 years and BMI z-score of 2.1 ± 0.3 at entry. Based on a cut-off score of 10 or higher on the SMFQ [42], 21 adolescents were classified as having depressive symptoms at entry. Moderate to extremely severe levels of symptomatology were reported for depression (score > 6), anxiety (>5), and stress (>9) in 20, 18, and 33 parents, respectively.

### Relationships between adolescent and parent psychosocial outcomes

Adolescent qualities of life (psychosocial, physical, and health-related) were negatively associated with parent symptoms of depression (health-related rho = −.22), anxiety (health-related rho = −.16), and stress (health-related rho = −.20), whereas adolescent symptoms of depression were positively related to parent symptoms of depression (rho = .17) and stress (rho = .18) (see Table 1). All correlations presented were statistically significant at p-value less than .05. Family functioning was not significantly related to adolescent psychosocial outcomes.

**Table 1:** Overall Spearman Correlations Between Adolescent and Parent Psychosocial Outcomes Across All Assessment Periods

		Adolescent			
		SMFQ	PsySoc	Phys	HRQL
Parent	D	.17^b^	-.20^b^	-.22^c^	-.22^c^
	A	.10	-.14^a^	-.14^a^	-.16^a^
	S	.18^b^	-.19^b^	-.17^b^	-.20^b^
	FF	.08	-.09	-.01	-.07

Note. *SMFQ* Short Moods and Feelings Questionnaire; *PsySoc* psychosocial quality of life; *Phys* physical quality of life; *HRQL* health-related quality of life; *D* Depressive symptoms; *A* Anxiety symptoms; *S* Stress symptoms; *FF* Family Functioning

^a^Correlation is significant at the 0.05 level (2-tailed)

^b^Correlation is significant at the 0.01 level (2-tailed)

^c^Correlation is significant at the 0.001 level (2-tailed)

### Changes in adolescent and parent psychosocial outcomes

Changes in adolescent psychosocial outcomes are shown in Table 2. Depressive symptoms were statistically lower at pre-intervention compared to baseline (mean difference of −1.3, 95 % CI: −2.5, −0.05, *p* = .042) and at 6 months compared to pre-intervention (mean difference −1.7, 95 % CI: −2.9, −0.5, *p* = .004).

**Table 2:** Changes in Adolescent and Parent Psychosocial Outcomes Across Study Periods

	Point Estimates						Rate of Change		
	*Mean (SE)*						*Mean Δ per month (95 % CI)*		
Variable	Baseline	Pre	Post	3 months	6 months	12 months	Waitlist	Intervention	Maintenance
							*(Baseline to Pre)*	*(Pre to Post)*	*(Post to 12 months)*
	Adolescent								
SMFQ	8.3 (0.4)*	7.0 (0.4)	6.4 (0.4)	6.0 (0.6)	5.3 (0.5)*	5.8 (0.7)	−0.4 (−0.8, −.002)	−0.3 (−0.9, 0.3)	−0.1 (−0.2, 0.1)
PsySoc	66.3 (1.3)	68.9 (1.2)	73.2 (1.3)*	77.8 (1.8)*	78.7 (1.7)*	74.6 (2.1)*	0.9 (−0.4, 2.1)	2.2 (0.4, 3.9)	0.1 (−0.3, 0.5)
Phys	72.4 (1.2)	73.9 (1.3)	79.1 (1.4)*	82.6 (1.5)*	84.8 (1.5)*	79.2 (2.4)	0.5 (−0.7, 1.7)	2.6 (0.7, 4.5)	.01 (−0.4, 0.5)
	Parent								
D	6.5 (0.5)*	5.1 (0.4)	5.6 (0.4)	5.4 (0.7)	5.1 (0.7)	4.7 (0.6)	−0.5 (−0.9, −.04)	0.3 (−0.4, 0.9)	−0.1 (−0.2, .04)
A	5.2 (0.3)*	3.4 (0.4)	3.6 (0.3)	3.1 (0.4)	3.8 (0.6)	3.3 (0.5)	−0.6 (−0.9, −0.2)	0.1 (−0.4, 0.6)	-.02 (−0.1, 0.1)†
S	11.9 (0.6)*	9.9 (0.6)	10.0 (0.6)	9.0 (0.7)	8.9 (0.9)	8.4 (0.8)	−0.7 (−1.2, −0.1)	0.1 (−0.8, 0.9)	−0.1 (−0.3, .02)
FF	1.7 (.05)*	1.5 (.04)	1.6 (.05)	1.6 (.05)	1.5 (.05)	1.4 (.05)	-.04 (−0.1, −.001)	0.1 (−.01, 0.1)†	−0.2 (−.03, −.01)

Note. *SMFQ* Short Moods and Feelings Questionnaire; *PsySoc* psychosocial quality of life; *Phys* physical quality of life; *D* Depressive symptoms; *A* Anxiety symptoms; *S* Stress symptoms; *FF* = Family Functioning

*significant difference from pre point estimate (*p* < .05)

†significant difference in the rate of change compared to waitlist period (*p* < .05)

Scores for psychosocial (mean difference 4.3, 95 % CI: 0.8, 7.8, *p* = .015) and physical (mean difference 5.2, 95 % CI: 1.6, 8.9, *p* = .005) quality of life were significantly improved from pre-intervention levels following the intensive 8-week intervention and remained improved for the majority of assessments during the 12-month maintenance period.

During the waitlist period from baseline to pre-intervention, significant reductions were shown for parent symptoms of depression (mean difference −1.4, 95 % CI: −2.7, −0.06, *p* = .040), anxiety (mean difference −1.7, 95 % CI: −2.8, −0.6, *p* = .002), and stress (mean difference −2.0, 95 % CI: −3.7, −0.4, *p* = .015) (see Table 2). There were no significant changes in parent reported depression, anxiety, or stress following the intervention or during the maintenance period, except monthly rates of change for anxiety symptoms were significantly increased (*p* = .004) compared to rates of change during the waitlist period, indicating an increase in anxiety symptoms during the maintenance period. The monthly rate of change in family functioning during the intervention period (0.1, 95 % CI: −.01, 0.1) also significantly increased (*p* = .026) compared to the rate of change during the waitlist period (−.04, 95 % CI: −0.1, −.001), indicating a slight deterioration in family functioning during the intervention. Sensitivity analyses conducted using participants who completed 12-months post-intervention assessments (*n* = 34) revealed consistent findings for all adolescent and parent psychosocial measures.

### Relationships between adolescent psychosocial outcomes and adolescent behavioural and physical outcomes

Analyses revealed participants with more positive changes in psychosocial quality of life had significantly greater increases in moderate to vigorous physical activity (mean difference 11.6 min/day, *p* = .017), and those with more positive changes in physical quality of life had significantly greater reductions in junk food consumption (mean difference −4.5 serves/day, *p* = .016), with no significant differences in behaviours demonstrated in relation to depressive symptoms (see Table 3). No statistically significant differences were demonstrated in physical outcomes between participants who reported improvements in psychosocial outcomes (psychosocial and physical quality of life and depressive symptoms) and those who did not report improvements at either post-intervention or 12-months following the intervention.

**Table 3:** Relationship Between Psychosocial, Behavioural, and Physical Changes by Tertile, estimated means (SD)

	SMFQ tertiles			PsySoc tertiles			Phys tertiles		
	Most positive change	Least positive change	*P*-value comparing tertiles	Most positive change	Least positive change	*P*-value comparing tertiles	Most positive change	Least positive change	*P*-value comparing tertiles
Change in SMFQ	−4.45 (2.81)	5.29 (4.34)	<.001	−3.0 (4.6)	1.6 (5.7)	.022	−3.7 (3.8)	0.6 (6.6)	.058
Change in PsySoc	8.1 (13.8)	−2.9 (11.6)	.018	21.5 (8.2)	−7.3 (7.1)	<.001	16.1 (11.4)	−0.8 (15.5)	.004
Change in Phys	12.5 (18.9)	−1.3 (10.9)	.018	18.0 (20.1)	−1.1 (12.8)	.002	27.8 (12.9)	−7.6 (9.0)	<.001
Change in BMI	0.17 (0.96)	0.36 (1.05)	.591	0.0 (1.2)	0.4 (1.3)	.434	0.1 (1.4)	0.3 (1.1)	.735
Change in BMI z-score	−0.025 (0.08)	−0.014 (0.08)	.676	-.001 (0.1)	-.006 (0.1)	.927	.01 (0.1)	−0.2 (.08)	.437
Change in MVPA (min/day)	6.36 (12.26)	3.86 (13.08)	.655	11.6 (8.2)	.007 (10.6)	.017	7.5 (9.7)	8.0 (13.3)	.932
Change in Junk (serves/day)	−2.25 (4.06)	−0.36 (3.61)	.239	−2.5 (3.2)	−1.5 (3.4)	.480	−5.9 (2.2)	−1.4 (3.4)	.016

Note. *SMFQ* Short Moods and Feelings Questionnaire; *PsySoc* psychosocial quality of life; *Phys* physical quality of life; *BMI* body mass index; *MVPA* moderate and vigorous physical activity

## Discussion

The primary aim of the current study was to examine the relationships between adolescent and parent psychosocial outcomes, with secondary aims exploring the changes in adolescent and parent psychosocial outcomes and relationships between adolescent psychosocial outcomes and behavioural and physical outcomes during participation in a community-based multi-disciplinary family lifestyle intervention. Results revealed that adolescent and parent psychosocial outcomes were related throughout participation, improvements occurred prior to actual participation and were mainly maintained at one-year follow-up, and adolescent psychosocial outcomes were partially related to behavioural outcomes and fully independent of physical outcomes.

### Relationship of adolescent and parent psychosocial outcomes

The study demonstrated significant associations in the predicted directions between adolescent and parent psychosocial outcomes, except for family functioning. Previous research indicates that psychosocial adjustment in youth seeking treatment is associated with parental psychological distress [43], yet no prior studies have reported on the association of adolescent and parent psychosocial outcomes for a minimum of one-year follow-up in a family-based healthy lifestyle intervention. The strength of association demonstrated between adolescent and parent psychosocial outcomes in the current study (rho = .17 to .22) is similar, and even stronger in some instances, to relationships previously demonstrated between adolescent psychosocial outcomes and factors postulated to contribute to obesity (i.e., screen time, physical activity, healthy eating) [44], suggesting that parent psychosocial outcomes may be just as significant, if not a more important variable, to consider in clinical practice.

The lack of association with family functioning scores was however surprising and may have been due to non-linear patterns of change sometimes demonstrated when navigating the implementation of new behaviours that challenge previous experiences [45, 46]. In contrast to parents’ potential challenges felt in implementing changes, adolescents may have still interpreted parental efforts as supportive. Despite differences in regard to family functioning, findings from the current study suggest parent psychosocial outcomes may be related to adolescents’ psychosocial outcomes with intervention participation and one-year follow-up. Although this relationship is likely to be bi-directional, due to high attrition it was not possible to examine the direction of the relationship. A key implication of the cross-sectional findings from the current study is that interventions targeting adolescents who are obese need to also address the psychosocial issues of their parents.

### Adolescent and parent psychosocial outcomes

Overall, participation in CAFAP was associated with a reversal in the trajectory of depressive symptoms previously demonstrated in adolescents who are overweight and obese [47]. These findings add to the previously reported psychosocial benefits (e.g., self-esteem) [18, 48] demonstrated in the context of a family-based multi-disciplinary intervention, by extending psychosocial outcomes to include maintenance of reductions in depressive symptoms at one-year follow-up. Although this is the first study to report on depressive symptoms in this context, the magnitude of change in mean scores demonstrated in the current study were similar to those reported in previous interventions targeting adolescent obesity [49].

It is interesting that depressive symptoms improved over the waitlist period, suggesting that changes may occur in adolescents’ psychological symptoms if they perceive impending availability of support. Fontaine et al. [50] suggest that intervention outcomes may be influenced via placebo-related factors such as expectations of improvement, interaction with staff, and desires of improvement. Indeed, in psychotherapy settings where participant expectations have been extensively studied, researchers have noted the importance of considering the impact of expectations on participant outcomes following intervention [51, 52]. A shift from enrolment (baseline) to intervention commencement (pre-intervention) may have restored participants’ hopes and positive expectations, and hence impacted on a reduction in symptoms [53]. Nevertheless, findings from the current study demonstrate the usefulness of participation in the intervention to maintain changes associated with potential expectations.

In addition to depressive symptoms, adolescent psychosocial and physical quality of life improved, and these changes were generally sustained up to 12-months post-intervention. The magnitude of changes in mean scores across intervention and maintenance periods were also consistent with the sparse literature available on sustained changes in quality of life outcomes in adolescents who are overweight and obese following participation in family-based interventions [14, 54].

Similarly, and surprisingly, significant reductions in parent depression, anxiety, and stress symptoms were demonstrated during the waitlist period rather than following the intervention. It is possible that psychosocial outcomes for parents may have been linked with a sense of empowerment related to taking action and seeking support for managing their adolescents’ overweight issue [55]. Parents often feel judged by others and report experiencing a sense of guilt and shame regarding their adolescents’ overweight status [56]. It is interesting that parents’ likely perceptions of impending support was substantial enough to contribute to significant reductions in symptomatology during the waitlist period. However, changes in mean scores maintained over the one-year period are consistent with the magnitude shown for interventions promoting healthy lifestyle behaviours in adults [57, 58] and suggests the importance of actual intervention participation.

The reductions in reported family functioning during the intervention suggest that family dynamics were likely evolving during these periods to reflect varying states of uncertainty experienced by adolescents and their parents [59, 60]. During the waitlist period adolescents and parents may have felt a sense of relief in relation to impending support leading to improved perception of family function. However once the intervention started, the realities of dealing with changing behaviours and challenges in parent-adolescent communication are likely to have highlighted family function difficulties. Previous investigations of parental expectations suggest that parents are likely to perceive intervention as resolutions to reducing current difficulties associated with their adolescents’ obesity and tend not to ascribe potential negative experience to implementing behaviour changes [61], which may predispose parents to experiencing a mismatch between expectations and reality. A positive aspect of the findings are that family function did not continue to deteriorate, but that providing a supportive environment for parents and adolescents enabled adaptive adjustments. Among the limited studies reporting on family functioning within the context of family-based interventions, outcomes have been mixed in both child and adolescent samples [62]. Findings from the current study suggest that previously reported inconsistencies in family functioning outcomes may be attributed to non-linear patterns of change overtime that reflect familial adjustment to changing dynamics.

### Adolescent psychosocial, behaviour and physical outcomes

It was anticipated that changes in psychosocial outcomes would be associated with changes in behaviours and physical status, however only changes in psychosocial and physical quality of life were related to improvements in moderate to vigorous physical activity and reductions in junk food intake, respectively. Although evidence suggests a relationship between quality of life and behavioural outcomes [63], this relationship has not been previously investigated in a family-based multi-disciplinary intervention with minimum of one-year follow-up [64]. The mean improvement of 11.6 min in moderate to vigorous physical activity per day demonstrated between adolescents in the higher and lower tertiles of psychosocial quality of life is nearly double the amount typically demonstrated in previous intervention studies [65]. In addition, a reduction of 4.5 junk food serves per day shown between adolescents in higher and lower tertitles of physical quality of life is greater than changes in similar measures of caloric intake shown in previous interventions [15]. It is possible that the association between psychosocial quality of life and moderate to vigorous physical activity is a response to adolescents engaging in physical activity with peers as a consequence of feeling more accepted. In comparison, personal perceptions of abilities to complete physical tasks may be a more salient factor in predicting one’s choice to improve healthy eating behaviours. Both relationships highlight the clinical relevance of considering psychosocial changes in healthy lifestyle interventions. Given the paucity of studies exploring this relationship in the current intervention context, future research would do well to further explore changes in qualities of life in relation to behavioural outcomes.

The limited association between depressive symptoms and behaviour outcomes was surprising given prior evidence in support of a relationship between these variables [66]. However, this is the first study to assess the relationship of these changes in adolescents following a multi-disciplinary intervention with one year follow-up [64]. A possible explanation for the limited association between changes in depressive symptoms and behavioural and physical outcomes is that in comparison to adolescent depressive symptoms, qualities of life may be more sensitive to the small effect sizes in behavioural changes demonstrated in the current study. Future investigations should examine whether limited changes in depressive symptoms persists in the presence of larger effect sizes in behavioural outcomes.

The limited association demonstrated between psychosocial and physical outcomes suggests that adolescents may experience improvements in psychosocial outcomes independent of changes in physical outcomes. Such independence of outcomes has been previously observed in relation to quality of life and weight changes following multi-disciplinary interventions [48], however, changes in depressive symptoms in relation to physical changes have not been previously explored in this context [64]. Findings from the current study suggest that pathways in predicting psychosocial outcomes may differ from those predicting behavioural and physical outcomes. Future research should therefore examine mediation effects between psychosocial, behavioural, and physical changes of interventions. In the interim however, an implication from the current study is that interventions for adolescents who are obese should specifically target psychosocial outcomes in addition to behaviour and physical outcomes.

### Strengths and limitations

This was the first study to examine the relationship between psychosocial outcomes of adolescents who are obese and their parents during participation in a family-based multi-disciplinary intervention, including one-year follow-up. While the ability to evaluate changes in psychosocial outcomes may have been weakened without a concurrent control group, the design was suited to the primary aim to explore the relationship between adolescent and parent outcomes, and the waitlist period with a double pre-test provided a within subject control comparison that was used to explore changes during participation. Generalizability may be limited by the relatively homogenous sample in one state of Western Australia and sample size due to high attrition. The rates of attrition and magnitude of changes are however similar to those demonstrated in other adolescent interventions and sensitivity analyses confirmed no differences in outcomes for the total sample and those who completed the entire study.

## Conclusions

The current study demonstrated a strong relationship between psychosocial outcomes in adolescents who are obese and their parents at both baseline and throughout participation in a family-based intervention that focused on the promotion of healthy lifestyle behaviours in a need-supportive context. Additionally, involvement in a family-based intervention positively impacted adolescents’ and parents’ psychosocial outcomes, and in some instances adolescent changes were related to changes in behaviour, but not physical status. The findings suggest interventions targeting adolescent obesity should: include parents and consider their psychosocial state, maximise the positive impact of families taking the initial action to engage with intervention, carefully support and manage family functioning during the change process by providing psychoeducation and normalising experiences, examine mediation in relation to psychosocial, physical and behavioural outcomes, and specifically target psychosocial outcomes independently of behaviour and physical outcomes.
